# Confidence level, challenges, and obstacles faced by orthopedic residents in obtaining informed consent

**DOI:** 10.1186/s13018-021-02531-1

**Published:** 2021-06-17

**Authors:** Abdulaziz Z. Alomar

**Affiliations:** Division of Arthroscopy & Sports Medicine, Department of Orthopaedic Surgery, College of Medicine, King Saud University, Riyadh, Kingdom of Saudi Arabia

**Keywords:** Informed consent, Residents, Orthopedic, Confidence level

## Abstract

**Objectives:**

The objective is to evaluate the opinions of orthopedic residents on current practices, experiences, training, confidence level, difficulties, and challenges faced when obtaining informed consent.

**Design:**

This is a cross-sectional, multi-center, and questionnaire-based study.

**Setting:**

The study was done in forty-four training centers across Saudi Arabia.

**Participants:**

In total, 313 orthopedic residents participated.

**Material and methods:**

The web-based questionnaire examined the perceptions of residents regarding practices, experience, training, difficulties, and challenges surrounding the obtention of informed consent, as well as residents’ confidence in obtaining informed consent for different orthopedic situations and eight common orthopedic procedures.

**Results:**

Most residents were allowed to obtain consent independently for all emergency, trauma, primary, and revision cases at their institution (92.7%). Only 33.5% of the residents received formal training and teaching on obtaining informed consent, with 67.1% having believed that they needed more training. Only 4.2% of the residents routinely disclosed all essential information of informed consent to patients. Inadequate knowledge (86.3%) and communication barriers (84.7%) were the most reported difficulties. Generally, 77.3% of the residents described their confidence level in obtaining informed consent as good or adequate, and 33.9% were confident to discuss all key components of the informed consent. Residents’ confidence level to independently obtain informed consent decreased with procedure complexity. Receiving formal training, senior level (postgraduate year (PGY) 4 and 5), and being frequently involved in obtaining informed consent correlated with increased confidence level.

**Conclusion:**

Many residents incompletely disclosed key information upon obtaining informed consent and reported lacking confidence in their ability to perform the procedure in their daily practices. To improve patient care and residents’ performance and overcome these difficulties and challenges, institutions should develop effective strategies to standardize the informed consent process, provide formal training for obtaining informed consent, and provide supervision for residents during obtention of informed consent.

## Introduction

Informed consent is a central component of patient-centered care; it involves physician-patient communication and requires effective communication skills to make patients aware of their clinical condition, to help them make decisions in the best interest of their health, hence being not just a sign on a piece of paper [1]. Surgeons have a legal and ethical obligation to inform patients about the details of the intended surgical intervention by conducting a valid informed consent process. For such consent to be valid, surgeons are required to answer all patient questions and necessitate to explain all important information related to the nature and course of the disease, the proposed surgical intervention, the surgery team and its members, the benefits of the procedure, the risks and potential complications of the procedure, the alternatives to surgery (including no surgery), and the outcome of illness without surgical intervention [2, 3].

Surgeons usually delegate the task of obtaining informed consent to residents, providing that they are trained, qualified, and have sufficient skills and knowledge [4, 5]. Furthermore, at most teaching institutions, junior residents are often the ones responsible for obtaining informed consent [6]. However, residents do not commonly receive structured formal teaching and training on how to obtain valid informed consent; instead, they often rely on informal learning (e.g., observance of their peers or seniors) to understand the process of obtaining of informed consent, and there is no consistent approach to teaching or evaluating the skills of residents regarding how to obtain a valid informed consent [7]. Studies have shown that information delivered to patients by physicians, particularly residents, is often inadequate [8–10]. The consequences of failing to adequately perform the proper process of acquiring valid informed consent can lead to poor physician-patient relationships, poor patient care and satisfaction, a suboptimal response to treatment, and even litigation [5].

Most previous studies exploring the informed consent process in the orthopedics field have focused on patient perspectives or documentations [11–14], so explorations of the residents’ perspective are currently lacking. Studies from different surgical specialties showed that residents experienced difficulty in obtaining informed consent and desired a training program in their curricula [15–18]. Moreover, although orthopedic residents are regularly responsible for obtaining informed consent, there is a general lack of data about their confidence in doing so and the challenges and barriers they face in such a process.

In English literature, no studies have focused on orthopedic residents’ perspectives on obtaining informed consent for surgical procedures. Therefore, this study aimed to explore the process by which residents obtain informed consent in the orthopedic field. Specifically, we were interested in the following topics from the residents’ perspectives: their experiences, training status, barriers and challenges, level of confidence in obtaining informed consent, and how the current informed consent process can be improved.

## Material and methods

This cross-sectional multi-center study recruited orthopedic residents at 44 training centers across Saudi Arabia. The inclusion criteria were (1) being orthopedic residents in a residency training program, (2) having at least 6 months of experience in orthopedics practice, and (3) having obtained informed consent for orthopedic procedures before being included in this study. All participants were allowed to fill in a web-based anonymous questionnaire that was designed to examine their perception of gaining informed consent, confidence level, and various difficulties encountered by them during the process. Residents from the enrolled centers who had active email accounts were invited to participate in an online survey using Google Drive.

The 57-item questionnaire comprised items on different areas related to informed consent collection, such as participants’ demographic data, opinions, experiences, trainings, practices, difficulties, and confidence level. The first component of the questionnaire involves items on demographics and participants’ opinions regarding the informed consent collection process. The second component involves items on their experiences and current practices related to the topic of interest. The third component focuses on the current learning and training methodologies and status for obtaining informed consent. The fourth component focuses on the barriers and difficulties encountered in the process. The fifth component asks about their confidence level in obtaining informed consent.

For items inquiring about confidence level, to ensure coverage of the most commonly performed orthopedic procedures in all subspecialties, eight surgical procedures were included: (1) intramedullary nailing of a femoral fracture, (2) total knee arthroplasty, (3) anterior cruciate ligament reconstruction, (4) discectomy, (5) open reduction for developmental dysplasia of the hip, (6) hallux valgus correction, (7) rotator cuff repair, and (8) osteosarcoma resection. The items also asked respondents to rate their confidence level in different surgical situations, such as emergency and trauma, elective primary simple, elective primary complex, and elective revision cases.

The questionnaire was responded on a 5-point Likert-type scale, with options ranging from strongly disagree to strongly agree, from not confident to strongly confident, and nine questions were closed questions with multiple answers. Additionally, there were four open-ended questions about the difficulties, challenges, and suggestions on how to overcome the difficulties faced by residents while obtaining informed consent. The study was approved by the King Saud University Institutional Review Board (Approval date 08.09.2020/IRB No. 20/0448).

### Statistical analysis

Categorical variables were summarized as percentages and compared using chi-squared test. Subsequently, post hoc analyses using adjusted residual values were performed to interpret the strong association between subgroups. To simplify, we divided study participants into a junior year group (PGY 2 and PGY 3) and a senior year group (PGY 4 and PGY 5) and sub-analyzed their data. Study participants were further divided based on their responses on the 5-point Likert-type scale into the agree group (respondents who chose “strongly agree” and “agree”), the neutral group, and the disagree group (respondents who chose “strongly disagree” and “disagree”). They were also divided by their confidence level into the confident group (respondents who chose “completely confident” and “fairly confident”), the neutral group (somewhat confident), and the not confident group (respondents who chose “not confident” and “slightly confident”). For correlations between study variables and confidence levels, Kendall’s tau-b (τb) coefficient has been used as a nonparametric measure of the strength and direction of the correlation (positive or negative).

The SPSS software, version 23 (SPSS Inc., Chicago, IL, USA), was used for data entry and statistical analysis. Microsoft Excel was used for graphical illustration. All analyses were performed with a significance level set to 0.05.

## Results

### Demographics and opinions of the study population

The questionnaire was sent out to 450 residents through email, of which 365 residents replied (response rate: 81.1%). Of these 365, 52 were PGY 1 residents (10.9%); these were excluded from the study because they were still undergoing core surgical rotations, hence having had not yet started their training in orthopedics and no experience in obtaining informed consent for orthopedic procedures (off-service rotations). Therefore, our final study sample comprised responses from 313 residents.

In total, the final sample comprised 274 (87.5%) men and 39 (12.5%) women. The PGY group distribution was as follows: 85 PGY 2 residents, 82 PGY 3, 83 PGY 4, and 63 PGY 5. Most residents (95%) agreed that the main purpose of the informed consent was for medical/legal documentation, to inform the patient, improve patient care, and protect physician liability (Table 1). Moreover, 70% of the residents disagreed that the informed consent should only be obtained by the surgeon performing the procedure or by a board-certified person. Meanwhile, 294 (93.9%) residents believed that nurses and other qualified health workers in the ward should not obtain informed consent from patients.

**Table 1:** Participants’ demographics and baseline characteristics (*n* = 313)

Characteristics		N (%)
**Sex**	Male	274 (87.5)
	Female	39 (12.5)
**Years of training**	PGY 2	85 (27.2)
	PGY 3	82 (26.2)
	PGY 4	83 (26.5)
	PGY 5	63 (20.1)
**Program region**	Central	118 (37.7)
	Eastern	68 (21.7)
	Southern	25 (8.0)
	Western	98 (31.3)
	Northern	4 (1.3)
**In your opinion, what is/are the main purpose(s) of the informed consent?**	Legal obligation to inform the patient	22 (7)
	Ethical obligation to inform the patient	5 (1.6)
	Improve patient care	3 (1)
	To protect physician liability	4 (1.3)
	All the above	297 (94.9)

*PGY* post graduate year

### Informed consent training for residents

Only 83 (26.5%) residents received guidelines from their institution about gaining informed consent; only 67 (21.4%) received adequate training in obtaining informed consent during their residency training; and 210 (67.1%) believed that they needed more training on how to obtain a valid informed consent. Most residents (*n* = 190; 60.7%) agreed that training on the topic should be conducted both formally and informally. Most (78.8%) agreed that formal training and protected teaching time on how to obtain informed consent was necessary.

**Fig. 1:**
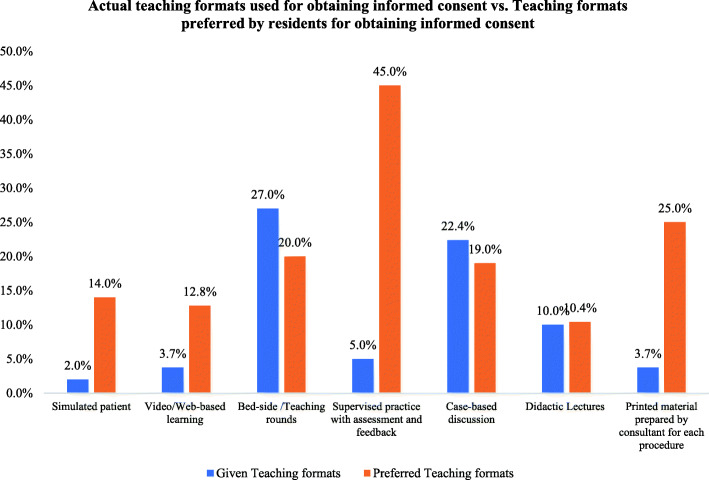
Bar chart of the percentages for actual teaching formats for informed consent training and the preferred teaching formats for residents

Importantly, only one third of the residents (*n* = 105, 33.5%) received formal training and teaching in obtaining informed consent (Table 2). Among these 105, the commonly used teaching formats were bedside/teaching rounds (27.0%) and case-based discussions (22.4%). By comparing the results for the currently used formal teaching methods and those preferred by residents, significant differences could be observed; residents preferred training formats other than those currently used, such as supervised practice with assessment and feedback (45.0% vs. 5.0%), printed material prepared by consultants for each procedure (25.0% vs. 3.7%), simulated patients (14.0% vs. 2.0%), and video/web-based learning (12.8% vs. 3.7%; *p* < 0.05; Fig. 1).

**Table 2:** Results of the survey about the current training, teaching, and issues related to obtaining informed consent from patients for orthopedic procedures

Training issues for obtaining informed consent	Answers	N (%)
Did your institution provide you with guidelines about informed consent?	Yes	83 (26.5%)
	No	230 (73.4%)
I have received adequate formal training in obtaining informed consent during my residency training	Strongly agree and agree	67 (21.4)
	Neutral	31 (9.9%)
	Strongly disagree and disagree	215 (68.6%)
I need more training on how to obtain a valid informed consent.	Strongly agree and agree	210 (67.1%)
	Neutral	78 (24.9%)
	Strongly disagree and disagree	25 (7.9%)
Teaching and training on how to obtain an informed consent should be conducted mainly by:	Only formal teaching	110 (35.1%)
	Only informal teaching	13 (4.15%)
	Both	190 (60.7%)
Formal training and protected teaching time on obtaining informed consent is necessary.	Strongly agree and agree	275 (87.8%)
	Neutral	25 (8%)
	Strongly disagree and disagree	13 (4.2%)
Did you receive FORMAL training/teaching in obtaining informed consent?	Yes	105 (33.5%)
	No	208 (66.5%)
The INFORMAL learning methodology of observing peers or seniors is inadequate and suboptimal.	Strongly agree+ agree	225 (71.9%)
	Neutral	13 (4.2%)
	Strongly disagree + disagree	75 (24.0%)

Additionally, 65% of the residents believed that it would be beneficial to use a form of decision support tool when obtaining consent, for example, diagrams, websites, DVD, or any information booklets.

When residents were asked about the most helpful informal learning formats, the most chosen options were as follows: observance of their senior or consultant while obtaining informed consent (*n* = 255, 81.5%), observation by senior students or peers of their performance in the procedure (*n* = 220, 70%), and informal discussions with their consultants or seniors on the topic (*n* = 190, 60.7%).

Regarding the open-ended questions on the suggestions to improve their skills in obtaining informed consent, the top three answers were obtaining informed consent at the OPD or during the round with the consultant (20.7%); having protected time for structured formal teaching and training (16.6%); and engaging in supervised practice by a senior person, especially in the junior years or complex cases (14.8%; Table 3).

**Table 3:** List of suggestions provided by residents on how to improve a resident’s skills in obtaining informed consents

What do you suggest to improve a resident’s skills in obtaining an informed consent?	N (% out of 169)
Obtaining informed consent at OPD or during the round with the consultant	35 (20.7)
Protect time for structured formal teaching and training	28 (16.6)
Supervised practice by a senior person, especially for junior level or complex cases	25 (14.8)
Handout materials for each procedure covering all information needed to be explained to the patient	17 (10.1)
Using any forms of decision support tools when obtaining informed consent from patients, such as diagrams, websites, DVD, or any information booklets, can be helpful	15 (8.9)
Start training on the early years of residency (junior level)	12 (7.1)
Language translation support services for medical terminology	11 (6.5)
The development of clear guidelines for obtaining informed consent	10 (6)
A standardized consent form for each procedure including all required information that needs to be disclose to the patient	7 (4.1)
Improvements in communication between the surgeon and residents about the planned procedures	5 (3)
Reading regarding the specific cases and forms to improve knowledge	4 (2.4)

In total, 169 participants responded to this question

### Current practices and experiences related to obtaining informed consent

Most residents confirmed that they were the physician in charge of obtaining informed consent at their institutions and that they were always or most often involved in this procedure (*n* = 300, 96%). They also confirmed that they could obtain consent independently for all procedures (i.e., emergency and trauma, primary, and revision procedures) at their institution (*n* = 286, 91.4%). Moreover, 30% of the residents experienced more than five situations where patients rejected the informed consent, and 67% experienced more than five incidents where they felt that they were unable to make the patient understand the information related to the surgical intervention (Table 4).

**Table 4:** Results of the survey regarding the current practices and experiences of residents when obtaining informed consent from patients for orthopedic procedures

Current practices and experiences	Answers	N (%)
How frequently do you obtain informed consent?	Always	220 (70.3)
	Often	80 (25.6)
	Sometimes	9 (2.9)
	Occasionally	4 (1.3)
	Never	0
For which cases are you allowed to, independently, obtain an informed consent at your institution?	Trauma and emergency cases only	9 (2.9)
	Trauma and elective primary cases only	18 (5.8)
	Trauma, elective primary, and elective revision cases	286 (91.4)
	Not allowed	0
How many times has a patient refused to sign the informed consent handled by you?	Zero	117 (37.4)
	1 to 5	155 (49.5)
	6 to 10	32 (10.2)
	11 to 20	7 (2.2)
	More than 20	2 (0.6)
How many times did you feel unable to make the patient understand the information related to the informed consent?	Zero	9 (2.9)
	1 to 5	94 (30)
	6 to 10	112 (35.8)
	11 to 20	65 (20.8)
	More than 20	33 (10.5)

**Fig. 2:**
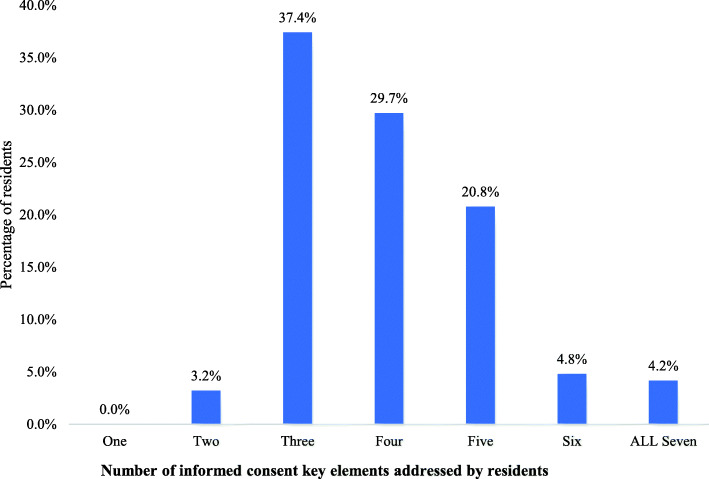
Bar chart of the percentage of residents versus total number of key elements of informed consents that they routinely discussed and addressed during obtaining informed consent

**Fig. 3:**
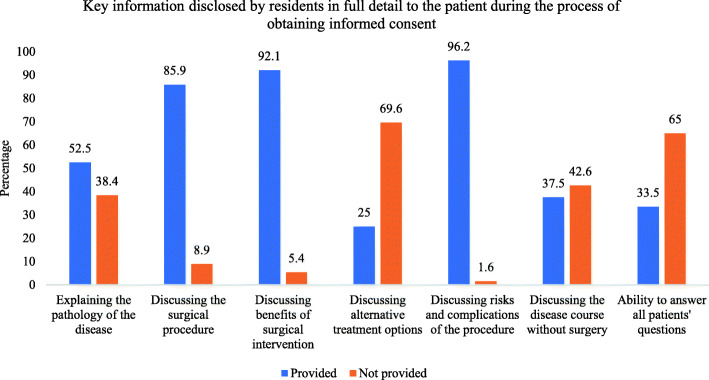
Bar chart of the ratio of participants that disclosed each of the seven key components of the process related to obtaining informed consent

Participants also responded about the information that they usually provided to the patient during the process based on the major key components of the informed consent, including (1) explaining the nature and course of the disease; (2) discussing alternatives to surgery; (3) discussing the surgical procedure and the surgical team and its members; (4) the benefits of surgery; (5) the risks and complications of surgery; (6) disease course without surgery; and (7) being able to answer all the patients’ questions. Despite the fact that all such aspects are key to the process, only 4.2% of the residents routinely discussed all of them; instead, most residents (37.4%) addressed only three of these key components (Fig. 2). Specifically, they usually discussed the risks and complications of surgery (96.2%), the benefits of surgery (92.1%), and the surgical procedures and the surgical team and its members (85.9%; Fig. 3).

### Barriers, difficulties, and challenges in obtaining informed consent

The major difficulties reported by residents were having inadequate knowledge (*n* = 270, 86.3%), communication and language barriers (*n* = 265, 84.7%), and the inability to answer all patients’ questions (*n* = 250, 79.9%) Table 5. On the items exploring which type of lack of knowledge was the major source of these difficulties, they reported that it was a lack of knowledge to explain about alternatives to surgery and about risks and complications of surgery. Regarding communication and language barriers, the most reported difficulty (*n* = 265, 84.7%;) was the translation of medical terminology to the local language (Arabic).

**Table 5:** Difficulties and barriers faced by residents when obtaining informed consent from patients for orthopedic procedures

	N (%)
**General barriers when obtaining informed consent**	
Inadequate knowledge	270 (86.3)
Communication and language barriers	265 (84.7)
Inability to answer the patient’s questions	250 (79.9)
Procedure complexity	220 (70.3)
High-risk consent	175 (55.9)
Level of patients’ education	132 (42.2)
Time constraints	130 (41.5)
Patients’ age	120 (38.3)
Urgency of the procedure	115 (36.7)
Dealing with the patient’s family (in pediatric and geriatric cases)	95 (30.4)
Cultural barriers	50 (16.0)
Patients’ sex	20 (6.4)
**Lack of knowledge**	
On alternatives to surgery	230 (73.5)
On risks and complications of surgery	210 (67.1)
On outcomes of non-treatment	122 (39.9)
On how much I should say to the patient	20 (38.3)
On the benefits of surgery	80 (25.6)
On how to describe the surgical procedure	30 (9.6)
**Communication and language barrier**	
Translation of medical terminology to the local language (Arabic)	265 (84.7)
Not being able to explain the surgery and its details in the local language	199 (63.6)
Proper documentation of informed consent in the Arabic language	180 (57.5)
Lack of interpreters	10 (3.2)

Survey question: “Which of the following do you think is/are considered as difficulties or barriers in obtaining an informed consent?”

### Confidence levels about obtaining informed consent

Residents were asked to rate their level of confidence in the following areas: in general, in discussing each key component of the informed consent, in different types of surgical situations in orthopedics, and on eight common procedures covering all orthopedic subspecialties.

**Table 6:** Results about residents overall descriptions regarding their general confidence level in obtaining informed consent from patients for orthopedic procedures

How would you describe your confidence level in obtaining informed consent?		N (%)
**Good**	I am confident and able to obtain valid informed consent in most situations, but I might need help in dealing with a particularly difficult patient or unusual set of circumstances	110 (35%)
**Adequate**	Under most circumstances, I can obtain adequate informed consent, but I can recognize problematic cases and seek help for complicated cases	132 (42.2%)
**Marginal**	I think my skills may be adequate for dealing with minor and common procedures and uncomplicated patients, albeit there might be deficiencies in more complicated situations. I might not always recognize such deficiencies and may fail to seek assistance when needed	51 (16.3%)
**Deficient**	I have concerns about my abilities to obtain adequate informed consent in most situations. I may fail to recognize my impaired capacity or exert undue influence on reluctant patients, potentially failing to meet adequate standards of disclosure	20 (6.4%)

Residents described their general confidence level in obtaining informed consent as good (*n* = 110, 35.1%), adequate (*n* = 132, 42.2%), marginal (*n* = 51, 16.3%), or deficient (*n* = 20, 6.4%; Table 4). There was a statistically significant difference between those who described their confidence level as good or adequate and those who described it as marginal or deficient (*p* value < 0.05) (Table 6).

**Fig. 4:**
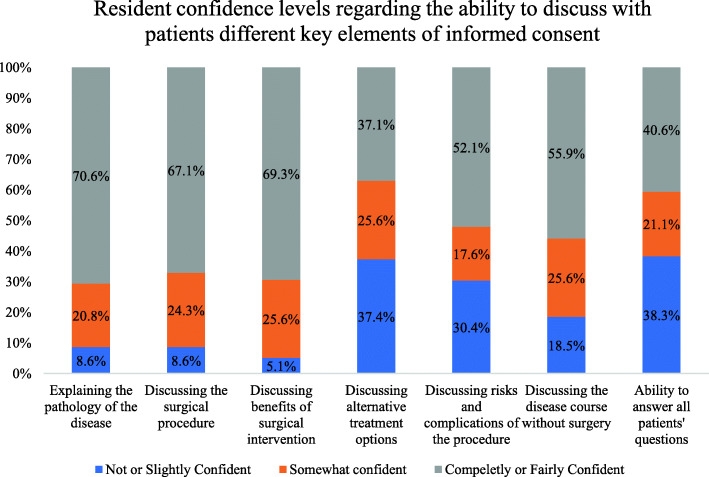
Bar chart of the confidence level of residents regarding the ability to discuss different key components of informed consent

Regarding their confidence in the seven key elements of informed consents (Fig. 4), residents were found to be significantly more confident in their ability to explain the nature and course of the disease (*n* = 221, 70.6%), discuss the surgical procedure and the surgical team and its members (*n* = 210, 67.1%), and discuss the benefits of surgery (*n* = 217, 69.3%) compared with those who were not confident (*p* < 0.05). Furthermore, the ratio of participants in the not confident group for discussing alternatives to surgery was significantly higher compared with that in the confident group (*p* < 0.05; Fig. 4). Only one third of the residents rated themselves as confident in addressing all seven components (*n* = 106, 33.9%).

**Fig. 5:**
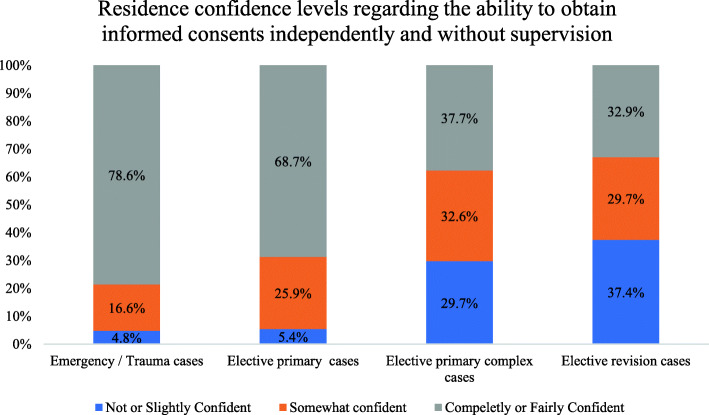
Bar chart of the confidence level of residents regarding the ability to obtain informed consent in different orthopedic cases independently

There were significant (*p* < 0.001) more residents in the confident group for emergency and trauma cases (78.6%) and elective primary cases (68.7%) compared with for elective primary complex cases (37.7%) and elective revision cases (32.9%). Moreover, the ratio of residents who felt that they needed assistance and supervision by a senior or consultant for obtaining informed consent was 84.0% in elective revision cases (*n* = 263), elective primary complex cases (*n* = 180, 57.5%), and elective primary simple cases (*n* = 80, 25.6%; Fig. 5).

**Fig. 6:**
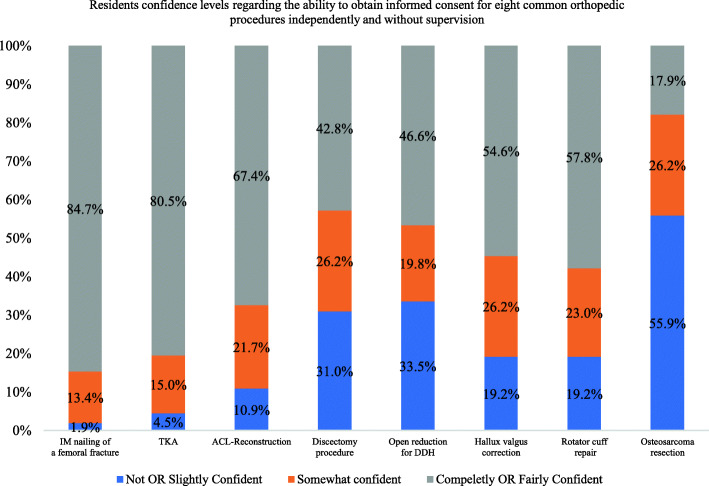
Bar chart of the confidence level of residents regarding the ability to obtain informed consent for eight common orthopedic procedures independently. IM: intramedullary nailing of a femoral fracture, ACL: anterior cruciate ligament reconstruction, DDH: developmental dysplasia of the hip

Regarding confidence in the eight common subspecialty procedures, a significant number of respondents described feeling confident (i.e., completely or fairly confident) in obtaining informed consent for intramedullary nailing of a femoral fracture (84.7%) and total knee arthroplasty (80.5%; *p* < 0.05). Meanwhile, a significant number of residents described feeling not confident (i.e., not or slightly confident) in obtaining informed consent for osteosarcoma resection procedures (55.9%; *p* < 0.05; Fig. 6).

### Factors correlated with confidence level for obtaining informed consent

**Fig. 7:**
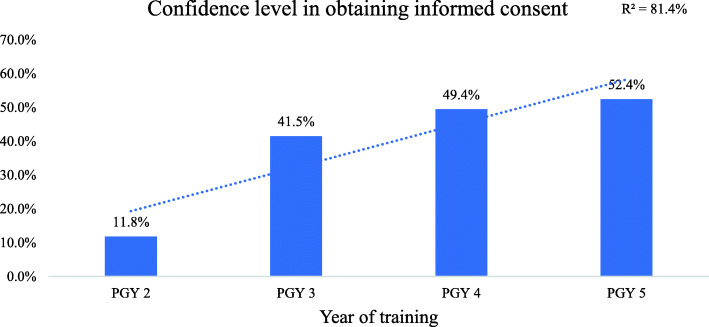
Bar chart and trendline for resident ratios who were “completely” or “fairly” confident in their ability to obtain informed consent from patients stratified by PGY. Data labels represent the percentages of confident participants in each group. PGY: postgraduate year. Survey question: “How would you describe your confidence level in obtaining informed consent?” *p* value for trend < 0.05

**Fig. 8:**
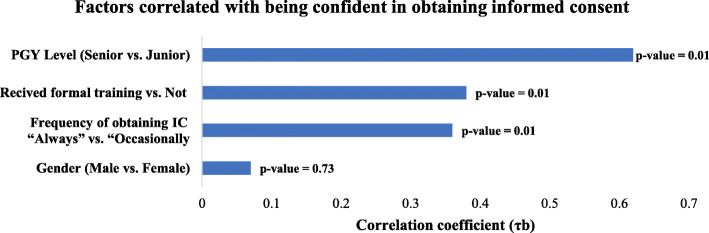
Bar chart representing the results of cross tabulation of Kendall’s Tau-b correlation coefficients (τb) between study variables and confidence levels for obtaining informed consent regarding all key components of informed consent. Numbers in table represent Kendall’s Tau-b correlation coefficients (τb). Numbers between brackets represent *p* values. NS: not significant. Senior PGY Group: PGY 4 and 5. Junior PGY group: PGY 2 and 3

We analyzed the correlation with four major factors: level of training, frequency of obtaining informed consent, residents’ sex, and the effect of receiving formal training and teaching. There was no statistically significant difference between male and female residents (77.0% vs. 79.5 %, *p* = 0.095). For level of training, there was a significant positive correlation between senior residents and junior residents (τb = 0.62, *p* value = 0.01); specifically, confidence levels increased significantly as residents advanced in level of training (Figs. 7 and 8).

Additionally, receiving formal training and teaching significantly correlated with a high confidence level (τb = 0.38, *p* = 0.01). The frequency of obtaining informed consent also had a positive correlation with confidence level, in that residents who were always involved in this procedure were more confident (τb = 0.36, *p* = 0.01; Fig. 8).

## Discussion

Orthopedics is a clinical branch considered to be at a high risk for claims, with one study from Italy having reported orthopedic surgeons to have been sued in 50% of cases [19]. However, most legal cases do not owe to failure in treatment but to failure in communication [5]. Studies have confirmed that a considerable proportion of lawsuit claims are not related to treatment errors, instead originating from improper conduction of the informed consent process, which can lead to miscommunication and misunderstandings [5, 19, 20].

Additionally, surgeons have been reported to often concentrate on the legal documentation of informed consent and neglect the disclosure and explanation of important information to the patient. Krause et al., exemplifying, found that the main reasons for patients to file lawsuit claims were discrepancies between the expected and achieved results of treatment (57.9%) and faulty information to patients (27.5%) [20]. An analysis of the lawsuits that included a claim of inadequate obtention of informed consent indicated that 17.9% of the case had residents involved in obtaining informed consent, as well as that properly conducted and documented informed consent was associated with a decreased risk of indemnity [19]. Therefore, effective informed consent obtention requires providing patients with adequate information, and the documentation of treatment decisions by the physician decreases litigation risks.

In this study, more than 95% of the residents agreed that the informed consent is essential, both legally and ethically, and that they are obligated to inform the patient through a valid informed consent process before any surgical procedure. However, 73% of the residents claimed that their institutions did not provide them with any specific guidelines regarding the legal aspects of informed consent; this finding was derived in the study despite the fact that the Ministry of Health (in collaboration with the Saudi Patient Safety Center) in Saudi Arabia has provided guidelines on what information should be shared with patients prior to them consenting to surgery [3]. Hence, it seems of vital importance for all medical institutions to provide a structured and standardized guidelines which potentially allowing residents to understand the importance of obtaining informed consent and apply it effectively in their practice.

The findings of this study showed that most of training centers rely on informal training, such as observing peers or seniors while obtaining informed consent or through informal discussions with consultants or seniors; additionally, only 33.5% of the residents reported that they had received formal training. Meanwhile, most residents agreed that informal training was inadequate and expressed interest in undertaking further formal training on obtaining informed consent. Most residents who received formal training were senior residents (PGY 4 and 5), and most who did not receive such training were junior residents (95%); this is despite junior residents being heavily and frequently responsible for obtaining informed consent. Based on the importance of informed consent, it seems imperative for training programs to include formal training on obtaining informed consents at an early stage, thereby ensuring that junior residents get exposed to this procedure and helping them develop confidence in the application of the process.

Based on prior research, residents are not specifically trained, and lack the competence, to guide patients through a legally correct informed consent process [5, 11–14]. Moreover, there seems to be no standardized/consistent approach for teaching informed consent skills. Few published studies have addressed how to train and improve residents’ skills in obtaining and documenting informed consent, such as using a SP [21, 22], case-based instruction [23], and an online module with a small group discussion with faculty [24]. In our study, bedside teaching and case-based discussions were the most commonly used formal teaching formats; however, residents preferred supervised practice followed by assessment and feedback, deeming it as the most effective way of training. They also believed that the most effective way of learning was at OPD or during the round with the consultant, especially for junior residents or when dealing with complex cases. Many participants also talked about the usability of pamphlets and pre-printed information sheets that could be specifically prepared for each procedure aiming to overcome the lack of knowledge which is considered as the major challenge related to the obtention of informed consent in this study.

When patients have to undergo surgery, surgeons are legally and ethically obliged to respect the patient’s autonomy by disclosing all details related to the surgical intervention in sufficient detail, giving patients the ability to make an informed decision. Nonetheless, the process of obtaining informed consent from and involving patients in clinical decisions is a complex, time-consuming, and challenging task for residents, and they also tend to receive little training on the topic. In the orthopedics specialty, the increasing number and complexity of surgical procedures, combined with the varying disclosure requirements, makes the process of obtaining informed consent potentially more difficult for residents. Nevertheless, orthopedics residents seem to be often requested in their residency training programs to provide information to and obtain consent from patients adequately and comprehensibly.

When obtaining informed consent for surgical procedures, physicians must explain not only the treatment proposed but also the alternative options to surgery, its benefits, complications, and the course of disease without surgery, needing also to make reasonable effort and provide appropriate time to answer all patient questions [2, 25]. Different studies have found that many physicians do not meet the minimum standards when they provide informed consent to patients, hence rarely discussing alternative treatment options, risks and complications, and benefits of the procedure [5, 26]. Meanwhile, residents were found to be more capable of informing the patient of the benefits of surgery than of informing about risks or alternatives [27]. Our findings were consistent with those of previously reported studies [22, 27], having confirmed that residents tend to not cover all important aspects related to obtaining informed consent. In our study, most residents explained to patients only the surgical procedures, the benefits, and the risk and complications of surgery. Meanwhile, most residents did not address alternatives to surgery and the course of disease without surgery and did not have the ability to answer all patients’ questions. Furthermore, only 4.2% of residents reported that they discussed all seven major elements of informed consents with patients. Such improper conducting of the process of obtaining informed consent could result in the violation of patient rights, specifically that related to the right of receiving sufficient information to conduct a well-informed decision-making regarding the choice of undergoing surgery.

Studies showed that residents frequently face situations in which they do not know what to tell a patient [27–30]. In our study, orthopedics residents ranked lack of knowledge as the major challenge related to the obtention of informed consent. As remarked previously, the great variety of subspecialties, surgical procedures, and techniques—which are applied differently depending on the nature of the problem, procedure details, benefits, risks, complications, expected outcomes, and alternative options—all mandate extra effort from residents to gain knowledge on each of the numerous procedures in medical practice. Several studies have demonstrated the deficiencies of residents in obtaining informed consent for specific procedures or treatments [7, 28, 29]. These deficiencies may be attributed to a variety of factors, including lack of awareness of procedural risks, poor communication skills in general, and lack of appropriate feedback on performance [28, 29]. Hence, it may be that lack of knowledge is a major burden for residents, one that can lead to failure in delivering appropriate information to the patient when they need to undergo surgical procedures. This may also explain why residents chose the use of printed materials—which should preferably be prepared by the attending surgeon for each procedure and cover all important related information needed for obtaining informed consent—as the second preferred learning format for obtaining informed consent.

Communication and language were also found to be major barriers for residents to obtain informed consents. Although all residents in our sample were Saudi and spoke the same mother language as patients (Arabic language), residents still ranked language barrier as the second major issue related to the obtention of informed consent. Moreover, the translation of medical terminology to the local language and the ability to explain the details of surgery in understandable language were deemed by residents as the most difficult barriers during the obtention of informed consent. A possible explanation for this is that all medical schools and training programs in Saudi Arabia teach medicine in English, making it a reasonable and expected inference that medical students and residents face challenges in communication when they try to explain medical topics to patients in the local language.

In total, 77% of the residents rated themselves as having good or adequate confidence in obtaining informed consent. To minimize the potential risk of inaccurate self-assessment and of over or underestimation, residents rated their confidence level for each element of informed consent. Indeed, the findings showed that residents overrated themselves, with only one third of the residents having rated themselves as confident in obtaining all seven elements of informed consent. This may indicate that residents are not aware of their limitations and suboptimal performance in the obtention of informed consent.

In contrast to general expectations, most medical lawsuits are generated after elective operations [14, 20]. In this study, we found that more than one third of the residents were not comfortable in obtaining informed consent independently, needing supervision for such procedure in elective complex primary and revision cases. Moreover, participants uniformly agreed that their level of confidence decreased as the complexity of the procedure increased; specifically, orthopedic oncological procedures (osteosarcoma resection) were deemed as the most challenging to explain, followed by a pediatric and spine-related procedures. Additionally, participants accepted that the main reason for refusal of consent was inadequate knowledge of the procedure; residents reported not being able to explain the risks and benefits of the procedure as well as the alternatives to surgery. Hence, the findings may justify the need for residents to obtain informed consent for complex procedures while being either supervised, or even for consultants to obtain it while residents observe the procedure.

Attending physicians have an ethical obligation to adequately supervise their trainees, ensure that they meet the minimal standard of care, and achieve a minimal competency level in communication skills needed to obtain a valid informed consent. The findings of this study showed that most junior residents rated themselves as slightly or not confident in obtaining informed consent independently in most situations, believing that they, instead, needed supervision. Given the prior discussions on most residents lacking knowledge on different aspects of interventions and the ability to answer patients’ questions, this low level of comfort in junior residents was somewhat expected. Houghton et al. found that 37% of junior physicians admitted to having already obtained informed consent for a procedure about which they had inadequate knowledge, and that a significant proportion of residents were not confident in or knowledgeable about obtaining informed consent [30]. Therefore, given that junior residents are supposed to be responsible for obtaining valid informed consent, there may be an urgent need for ensuring their accessibility to sufficient knowledge about the proposed surgical intervention and that they are suitably trained and supervised on this process.

This study found that receiving formal training to obtain informed consent was highly correlated with a high confidence level. Furthermore, residents who received formal training tended to conduct a more proper informed consent process by covering most of the key components of informed consent. Based on these findings, formal training on how to obtain informed consent seems to be the single important factor in improving residents’ confidence level and possibly improve their performance.

This study had some limitations. First, it did not attempt to assess residents’ skills and performance by independent assessors; therefore, the results of this study do not reflect how they performed and do not allow for assessing their skills. Second, the potential of over- or underestimation regarding confidence levels owing to the measurement tool having been self-assessed; hence, future research should examine the performance and confidence level of residents regarding the obtention of informed consent by direct observation, audit review, patient satisfaction, and patient understanding levels, among others. Third, the methodological approach used in this study does not allow for ruling out the potential of self-reporting bias, so the findings may not be completely accurate because some residents may have reported things that they actually do not perform.

## Conclusion

A significant percentage of residents are not accurately conducting the process of obtaining informed consent in their daily practice; they also seem to be lacking confidence in their ability and performing sub-optimally in conducting a valid informed consent process. To improve patient care and resident performance and overcome these difficulties and challenges, institutions and training programs should endeavor to develop effective strategies toward structuring and implementing formal training on how to obtain valid informed consent; they should also provide supervision for junior residents during the practice of this process. Furthermore, residents should be assessed in their competency to obtain informed consent before they graduate, ensuring that they are well-prepared to conduct this procedure prior to engaging in independent clinical practice. Finally, there is a need for further research on forms to improve the informed consent process and residents’ skills in it.

## Data Availability

All data related to this study are available and ready upon your request

## Abbreviations

ACL: Anterior cruciate ligament
DDH: Developmental dysplasia of the hip
IM: Intramedullary nailing
PGY: Postgraduate year
